# A Scoping Review of the Empirical Literature on Peer Support for People Living with HIV

**DOI:** 10.1177/23259582211066401

**Published:** 2021-12-17

**Authors:** Anita Øgård-Repål, Rigmor C. Berg, Mariann Fossum

**Affiliations:** 1 4343 University of Agder, Grimstad, Aust-Agder, Norway; 2 25563 Norwegian Institute of Public Health, Oslo, Norway, and University of Tromsø, Tromsø, Norway

**Keywords:** HIV, chronic disease, peer support, experiences, scoping review

## Abstract

People living with HIV receiving antiretroviral therapy need support related to linkage to care and self-management in everyday life. Peer support has been found to provide varied support according to the unique needs of the group. This scoping review aims to provide an overview of research on peer support provided to people living with HIV. A search was conducted in eight databases until May 2021, and two reviewers independently screened all identified studies. We sorted the included studies into categories and conducted descriptive analyses. For this communication, we included 34 studies representing three study categories: the experiences of peer support (n = 23), program descriptions (n = 6), and training of peer supporters (n = 5). The studies were published between 2000 and 2021 and included 4275 participants from 10 countries. The flexibility of peer support complements healthcare services, but there is a need to clarify and adjust the ongoing support when living with HIV.

## Background

With 37.6 million people living with human immunodeficiency virus (HIV) infection at the end of 2020, HIV remains a worldwide public health concern. Although global and national actions have halted and reversed the acquired immunodeficiency syndrome (AIDS) epidemic and reduced the overall incidence of HIV, the prevalence of HIV infection is still increasing in some countries and regions.^1^ Furthermore, antiretroviral therapy (ART) provision in highly endemic settings, such as sub-Saharan Africa, are challenged due to shortages linked to universal health coverage (Joint United Nations Programme on HIV/AIDS [UNAIDS].^2^ The Global Health Sector Strategy on HIV 2016-2021^3^ outlines a multisectoral response as a strategy that highlights the importance of involving the community, particularly people living with HIV [PLHIV], to effectively deliver health services.^3^

People from key populations, that is, those at elevated risk of acquiring HIV infection (including sex workers, people who inject drugs, prisoners, transgender people, and men who have sex with men) tend to have less access to ART and ordinary healthcare services.^4,5^ However, for PLHIV receiving ART, HIV has become a manageable chronic lifelong condition (CLLC).^1^ Unfortunately, since the beginning of the epidemic, HIV infection has been associated with social stigma and prejudice, and it remains one of the most stigmatized diseases in almost every culture worldwide.^6,7^ In addition, co-infections such as hepatitis, tuberculosis, and other comorbidities constitute an increasing burden among PLHIV,^3^ with noncommunicable diseases (NCDs) and mental health disorders as some of the most prevalent comorbidities.^3,8,9^

To manage the differentiated needs of PLHIV as described above, there is a need to prioritize specific populations and settings while providing HIV services.^10^ Peer support interventions have been highlighted as a flexible and promising approach to provide linkage to and adherence to ART among PLHIV.^10,11^ Peer support for PLHIV has a long history and grew out of the reactions of activists in the 1980s to combat stigma and discrimination. PLHIV still constitute communities of people experiencing stigma or fear of exposure and ostracization.^12^ The World Health Organisation (WHO) defines individualized peer support as “one-to-one support provided by a peer who has personal experiences of issues and challenges similar to those of another peer who would like to benefit from this experience and support.”^13^ (p.1). Dennis et al.similarly defined the concept of peer support as “the giving of assistance and encouragement by an individual considered equal.".^14^

Peer support is one way of involving patients to strengthen supportive resources in healthcare services and increase self-management,^11^ and diverse peer support models have been applied across various health contexts.^11,15,16^ Peer support from the larger HIV community is essential^12^ and has been found to reduce stigma.^17^ Peer supporters offer support and encouragement to their counterparts through meetings ranging from informal visits and shared experiences to formal appointments focused on practical information sharing. National standards for peer support in HIV were published in the UK to ensure that peer support is provided to PLHIV by PLHIV, and that peer support is tailored to the needs of PLHIV.^12^ A similar standard was recently published by the National Association of People With HIV Australia.^18^

More than a dozen systematic reviews of the effectiveness of peer support for PLHIV suggest that peer support is flexible enough to be applied across healthcare contexts and diverse populations,^5,19–21^ positively affect communities,^22^ and is a feasible and practical approach for linking and retaining PLHIV in HIV care.^23^ Unlike the numerous reviews investigating the effectiveness of peer support for PLHIV, few reviews exist on other aspects of this topic, such as the experiences of peers with peer support or the needs of peer supporters. Further, despite the conceptual analysis of peer interventions put forth by Simoni et al.^24^ and two reviews on providers’ perspectives of peer support,^15,25^ the scope of empirical research undertaken on peer support for PLHIV remains unclear and there is a need to map the rapidly expanding field of research on this topic. To this end, to better understand the scope of the current state of research and identify research gaps, this scoping review aimed to identify the characteristics of studies investigating peer support for PLHIV and the key results thereof.

## Methods

### Design

The present scoping review was conducted following the guidelines for scoping reviews.^26–28^ We report the results in accordance with the Preferred Reporting Items for Systematic Reviews and Meta-Analyses (PRISMA) extension for scoping reviews (PRISMA-ScR).^29^ The methods used in this scoping review, including its objectives and inclusion criteria, were specified in advance and documented in a published protocol (CRISTIN ID  =  X).

### Search Strategy for the Identification of Studies

Our preliminary searches in the Joanna Briggs Institute Database of Systematic Reviews and Implementation Reports and PROSPERO identified relevant reviews and keywords. We used population, concept, and context as our search framework because the research question implies that the context is not predefined.^30^ We searched in an online Medical literature Analysis and Retrieval System (MEDLINE) (OVID), MEDLINE In-Process (OVID), Embase (OVID), CINAHL (EBSCOhost), PsycINFO (OVID), SocINDEX (EBSCOhost), Social Work Abstracts (EBSCOhost), and BASE (Bielefeld Academic Search Engine) for the period 1981 to May 2021. Only papers published after 1981 were included, as this was the year in which studies on HIV/AIDS were first published. Our search strategy incorporated pre-specified subject headings and text words in the titles and abstracts adapted for each database. One reviewer (XX) conducted the search with an information search specialist, who was also consulted regarding the search strategy. The search strategy is presented in the supplemental material (Online Supp 1). In collaboration with the information search specialist, we searched for gray literature on Google Scholar, the UK government website, and COnnecting REpositories (CORE), a website that aggregates all open access research outputs from repositories and journals worldwide and makes them publicly available. In addition, we manually searched the reference lists of the included studies and relevant reviews and forward citation searches through the Web of Science (May 2021).

### Eligibility Criteria

Considering the aim of the review, the main inclusion criterion was studies that used empirical quantitative and/or qualitative research methods to address peer support among PLHIV. Both those who were receiving and providing peer support needed to be PLHIV aged 18 years and older. We followed the definition of peer support interventions/programs proposed by Dennis,^14^ whereby assistance and encouragement were obtained from an individual considered equal. Specifically, PLHIV had to use their own experiences of living with HIV to support other PLHIV through face-to-face interactions. Further, we considered studies ineligible if they were on children or youth, focused on primary prevention of HIV or mother-to-child transmission, or described PLHIV support groups. However, studies on mixed populations or interventions (eg, those including both adults and youth) were included if at least half of the population or intervention met the inclusion criteria or if the results were reported separately for our population and intervention of interest. We enforced no settings or publication format limits but included only publications in English or Scandinavian languages (Norwegian, Swedish, and Danish).

### Selection of Literature

We stored retrieved references in an Endnote X9 database (Thomas Reuters, New York, NY), deleted duplicate entries, and imported the references to the web-based software platform Rayyan.^31^ Using Rayyan, two reviewers independently screened all titles and abstracts according to the inclusion/exclusion criteria (XX, XX/XX). We promoted all relevant publications to full-text screening, which was independently performed by three reviewers. We attempted to retrieve the full texts of any studies that were unavailable in the public domain by contacting the main author. We resolved differences in opinion during the screening process at each stage through a re-examination of the study and subsequent discussion. Arbitration was achieved through discussion in consultation with a third reviewer.

### Data Extraction and Synthesis (Charting data)

Considering the aim of this review and the scope of scoping reviews in general, whereby methodological quality assessment is not a prerequisite, we did not appraise the included studies.^32^ One reviewer (XX) performed data extraction. Two other reviewers checked the completeness and accuracy of the data extracted from all studies and corrected the data when necessary. A predesigned and piloted data extraction form was used to ensure standardization and consistency.^32^ The data were extracted regarding author, year, study characteristics (eg, country, study design, and sample size), population characteristics (eg, gender, sexual identity), peer support characteristics (eg, term of peer support, duration, content, and settings), and main findings/results. We also categorized the interventions based on the four key functions of peer support described by Fisher et al. and the Peers for Progress program.^11^ Studies with unclear or minimally described intervention characteristics were not included. By keywording^33^ each study by such variables and compiling the data in a single spreadsheet, we could group them according to their main characteristics and conduct descriptive analyses using frequencies and cross-tabulations. The grouping included sorting the studies into clusters according to their relations to each other.^27,33^ Similarly, we copied the main findings of qualitative studies in a Word document, restricted to instances across the data with relevance to peer support, and looked for patterns. The results were summarized in the dataset.

## Results

**Figure 1.**
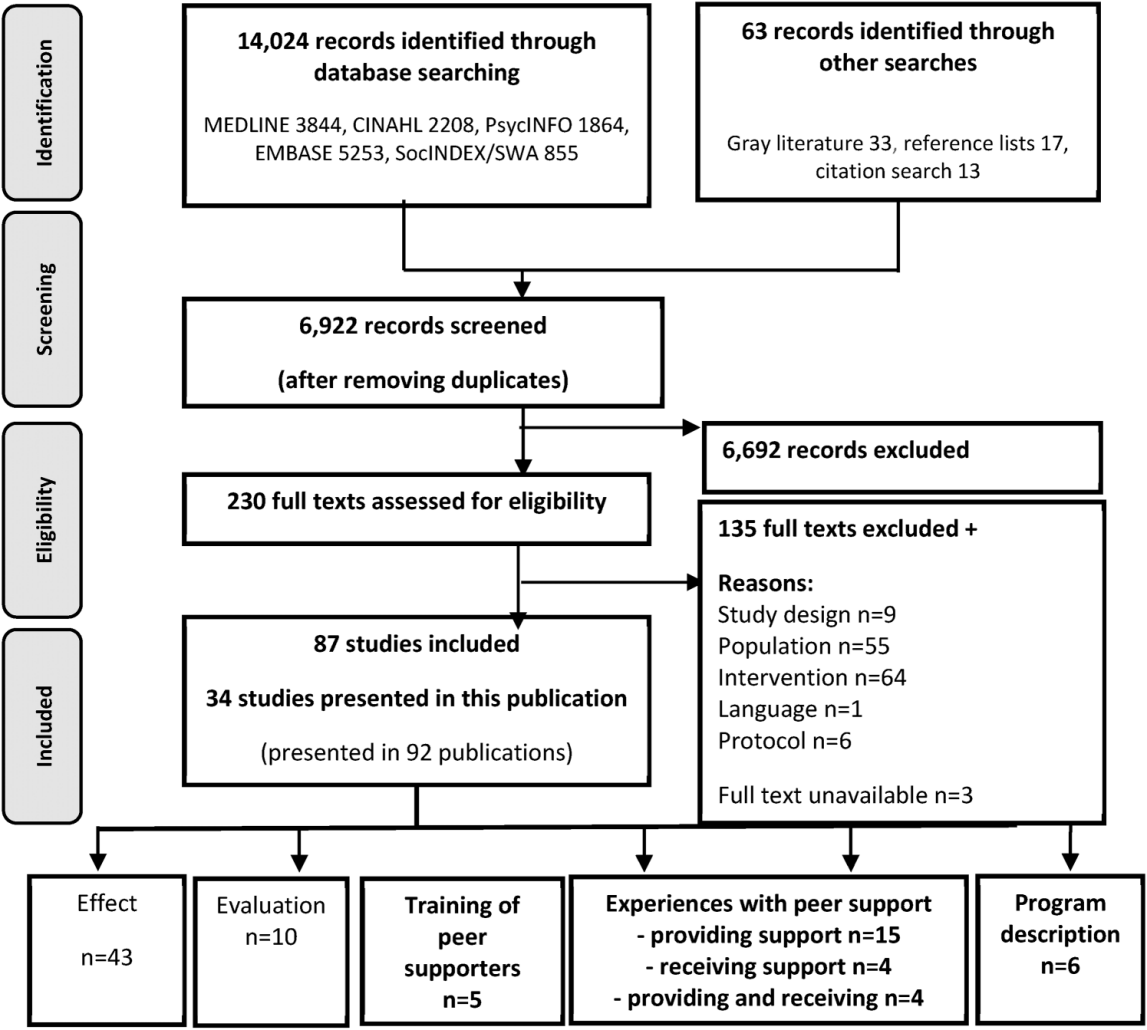
PRISMA flow diagram of the literature review process.

The searches resulted in 6922 individual records, of which 230 were considered potentially relevant (Figure 1).

Eighty-seven studies met the inclusion criteria. Due to the high number of included studies and the volume of data, it was necessary to separate the results into two reports. Our categorization of studies by objective/aim produced five categories of studies, which we used to separate the results into two reports. A description of the 53 studies that examined the effects of peer support and evaluations (implementation, process, feasibility, and cost) are available elsewhere.^34^ The present study addressed 34 studies that examined experiences with peer support (experiences providing and/or receiving peer support) (n = 23), program descriptions (n = 6), and descriptions of the training of peer supporters (n = 5) (Tables 1 and 2). Studies that fit more than one category were placed in the category that most closely matched the overall objective of the paper.

**Table 1.** Characteristics of the Included Studies (n = 34).

Study no.	Author, year	n	Country	Study design	Term/ label	Key function
**Experiences with peer support (n = 23)**						
1	Akinde et al. 2019	15	USA	Qualitative	Peer mentor	Assistance; Linkage
2	Alamo et al. 2012	347	Uganda	Mixed method	Community health worker	Assistance; Linkage
3	Born et al. 2012	230	Zambia	Mixed method	Peer educator	Assistance; Support; Linkage
4	Cane, 2018	6	England	Qualitative	Support worker	Ns
5	de Souza, 2014	31	India	Qualitative	Peer worker	Ns
6	Driskell et al. 2010	41	USA	Qualitative	Peer counselor	Assistance
7	Dutcher et al. 2011	23	USA	Qualitative	Peer educator	Ns
8	Enriquez et al. 2013	15	USA	Qualitative	Peer	Ns
9	Greene et al. 2015	121	Canada	Qualitative	Peer case manager	Assistance; Support; Linkage
10	Gusdal et al. 2011	118	Uganda and Ethiopia	Qualitative	Peer counselor	Ns
11	Harris and Alderson, 2007	12	Canada	Qualitative	Peer supporter	Ns
12	Harris and Larsen, 2007	12	Canada	Qualitative	Peer supporter	Ns
13	Houston et al. 2015	11	USA	Qualitative	Peer facilitator	Support
14	Kyakuwa, 2010	Ns	Uganda	Qualitative	Expert client	Assistance; Support; Linkage
15	Lee et al. 2015	12	South Korea	Qualitative	Peer supporter	Assistance; Support; Linkage
16	Li et al. 2015	27	Canada	Qualitative	Peer supporter	Ns
17	Mackenzie et al. 2012	68	USA	Qualitative	Peer mentor	Assistance
18	Marino et al. 2007	9	USA	Qualitative	Peer	Assistance; Support; Linkage
19	Messias et al. 2006^1^	6	USA	Qualitative	Peer counselor	Ns
20	Moyer et al. 2014	10	Kenya	Qualitative	Peer mentor	Assistance; Support; Linkage
21	Sunguti et al. 2019	230	Kenya	Descriptive	Peer educator	Assistance; Support; Linkage
22	Tan, 2012	21	USA	Mixed method	Peer	Ns
23	Tobias et al. 2010	186	USA	Cross-sectional	Peer	Ns
**Program descriptions (n = 6)**						
24	Karwa et al. 2017	1357	Kenya	Mixed method	Peer	Support; Linkage
25	Leonard et al. 2013	Ns	USA	Mixed method	Peer	Assistance; Support
26	Purcell et al. 2004	966	USA	RCT	Peer mentor	Assistance; Support; Linkage
27	Raja et al. 2007	122	USA	Mixed method	Peer	Assistance; Linkage
28	Tenthani et al. 2012	114	Malawi	Mixed method	Expert client	Linkage
29	Thomas et al. 2008	25	USA	Qualitative	Peer supporter	Ns
**Training of peer supporters (n = 5)**						
30	Allicock et al. 2017	6	USA	Mixed method	Peer	Assistance; Linkage
31	Cully et al. 2012	7	USA	Mixed method	Peer mentor	Ns
32	Kim and Shin, 2015	32	South Korea	Qualitative	Peer caregivers	Ns
33	Tobias et al. 2012	91	USA	Mixed method	Peer	Ns
34	Wolfe et al. 2013	4	USA	Mixed method	Peer	Linkage

1 This study was reported in multiple publications: see also Messias et al. 2009. Ns: not stated, PS: Peer support, RCT: Randomized controlled trial, Assistance: Assistance in daily management, Linkage: Linkage to clinical care and community resources, Support: Social and emotional support.

**Table 2.** Summary Characteristics of the Included Studies (n = 34).

Characteristics	All studies (n = 34)	Experiences (n** **=** **23)	Training (n** **=** **5)	Program description (n** **=** **6)
Year of publication				
**2015** to 2021	10 (29)	7 (30)	2 (40)	1 (17)
**2010** to 2014	17 (50)	12 (53)	3 (60)	2 (33)
**2005** to 2009	6 (18)	4 (17)		2 (33)
**2000** to 2004	1 (3)			1 (17)
**Country/setting**				
Canada	4 (12)	4 (17)		
Kenya	3 (9)	2 (9)		1 (17)
South Korea	2 (6)	1 (4)	1 (20)	
Uganda^1^	3 (9)	3 (13)		
**USA**	18 (53)	10 (44)	4 (80)	4 (67)
Other	4 (12)	3 (13)		1 (17)
**Study design**				
**RCT**	1 (3)			1 (17)
Qualitative	20 (59)	18 (78)	1 (20)	1 (17)
Mixed method	9 (26)	3 (13)	2 (40)	4 (66)
Other	4 (12)	2 (9)	2 (40)	
**Gender of participants**				
Male	4 (12)	3 (13)	1 (20)	
Female	3 (9)	3 (13)		
Male and female	19 (56)	14 (61)	3 (60)	2 (33)
Male, female, and transgender	2 (6)		1 (20)	1 (17)
Not stated	6 (18)	3 (13)		3 (50)

Legend: The ‘other’ countries were England, India, Malawi, Zambia.

1 One study was conducted in both Uganda and Ethiopia.

RCT: randomized controlled trial.

### Characteristics of the Included Studies

The main characteristics of the included studies are presented in Table 2. All studies were published in English. The number of publications on the topic of peer support for PLHIV has increased rapidly, from no publications prior to 2000 to only a few publications between 2000 to 2009 and 27 publications from 2010 to 2021. The study designs varied, but most were qualitative (n = 20) or mixed-method studies (n = 9). In addition, the study settings varied, but most studies were conducted in the United States (U.S.) (n = 18), while the fewest studies were conducted in Europe (n = 1). The total number of participants in the included studies was 4,275, with a majority of the studies including both men and women (n = 19); however, four studies included only males and three included only females as priority groups. Only two studies included non-binary-gender participants.

### Key Functions of Peer Support

Our results of the key functions of peer support^11^ demonstrated the different roles and key functions of peer support delivered across the studies (Table 1). The commonest key function of the intervention was linkage to clinical care and community resources (n = 15) and assistance in daily management (n = 15), followed by social and emotional support (n = 11). Several peer support interventions have a combination of the described functions. Notably, none of the included studies focused explicitly on ongoing support related to chronic diseases. In 15 (44%) studies, the description was too limited to categorize peer support interventions by key functions.

### Terms and Labels

In this set of 34 studies, we identified 12 different labels/names for peer supporters (Table 1). In the period 2000 to 2009, the terms “peer, peer counselor/supporter/mentor” were used. In the years 2010 to 2021, in addition to the labels used in prior years, a range of new labels appeared: “peer educator/worker/facilitator/case manager/caregiver”, “community health worker”, “support worker”, and “expert client”. The most frequently used label across the 34 included studies was “peer” (n = 10), followed by “peer supporter” (n = 5), “peer mentor” (n = 5), and “peer counselor” (n = 3).

### Categories of Studies and their Results

#### Studies about experiences

Of the 23 studies about experiences with peer support, 15 concerned experiences with *providing* peer support (Table 1; studies 2, 4, 5, 7-9, 15-23),^35–49^ four addressed experiences with *receiving* peer support (Table 1, studies 1, 6, 11, 13),^50–53^ and four explored PLHIV's views on both providing and receiving peer support (Table 1, studies 3, 10, 12, 14).^54–57^ Most studies (n = 20, 59%) utilized a qualitative design (Table 2). The four studies that covered experiences both with providing and receiving peer support included 360 participants in Uganda, Ethiopia, Zambia, and Canada. The results of these studies are combined with those of studies on experiences with providing and receiving peer support (below).

##### Experiences with providing peer support

Overall, the 15 studies on experiences with providing peer support comprised 1112 male and female participants from nine countries and 11 studies utilized a qualitative design. These studies on experience covered various peer support interventions. The studies varied in their main focus on experiences with providing peer support. A majority of the studies focused mainly on the role of peer supporters when meeting PLHIV. Other main interests were the challenges of being a peer supporter, their experience with the delivery of support, experiences with implementing peer support, and preferences concerning personal contact versus telephone support.

With respect to the results, nine studies reported that peer supporters provided practical, informational, emotional, and/or social support (studies 4, 5, 7, 9, 10, 12, 14, 17, 23)^36–38,40,43,49,55–57^ and modeled healthy behavior (studies 8, 9, 17).^39,40,43^ Studies have shown that peer supporters feel empowered in their own lives, have different motivations (such as being a role model and helping others), learn new skills and share knowledge, gain self-awareness, and become more visible in the community.^44,55–57^ Three studies described peer supporters as positive supplements to healthcare services. However, they noted the need to pay attention to issues such as work-related stress, training, and emotional suppor.t^35,41,51^

##### Experiences with receiving peer support

All four qualitative studies that explored experiences with receiving various types of peer support included 79 participants from the U.S. and Canada (studies 1, 6, 11, 13).^50–53^ The results indicated multiple benefits of meeting a peer supporter: a role model for living with HIV; social, informational, emotional, and instrumental support; and referrals to other care organizations that helped them connect with their community.

#### Studies presenting program descriptions

Six studies that included a total of 2584 participants used various data to describe a peer support program.^58–63^ (Table 1) Four of the studies were conducted in the U.S. (studies 25-27, 29),^59–61,63^ and three of these studies prioritized people of color (studies 25, 27, 29).^59,61,63^ Each of the six studies described a different program: an inpatient HIV peer navigator program which aimed to improve diagnosis and linkage to and retention in care (study 24),^58^ AIDS clinical trials (ACT) (study 25),^59^ Interventions for Seropositive Injectors Research and Evaluation (INSPIRE) (study 26),^60^ the Treatment Advocacy Program–Sinai for African Americans (study 27),^61^ an expert patient program in Malawi (study 28),^62^ and the Caribbean HIV Evaluation Support demonstration program (study 29).^63^ These focused equally on linkage to clinical care and community resources, assistance in daily management, and social and emotional support.

#### Studies on the training of peer supporters

The third and last category of studies covered five studies on the training of peer supporters (Table 1).^64–68^ All except one of these studies were conducted in the U.S. (study 32).^66^ Overall, there were 140 male and female participants in the five studies, of which one utilized a qualitative design and four used mixed methods. The peers varied in training. Two studies trained peer supporters in motivational interviews in peer support programs (studies 30, 34).^64,68^ One study tested a standardized training program for mentors in MAPPS (study 31),^65^ another developed a simulation-based training program for peer supporters who would care for terminally ill PLHIV (study 32),^66^ and the last study described a trainer program, which trained health educators and program directors (study 33).^67^ All five studies supported the value of and the need for quality training of peer supporters to ensure that peer supporters met performance standards.

## Discussion

Our scoping review, which aimed to provide an overview of the characteristics and results of empirical research on peer support for PLHIV, identified 34 studies published since 2000 on first-hand experiences with peer support, program descriptions, and depictions of the training of peer supporters.

Similar to the results of the 53 studies on the effects of peer support and evaluations, which we present elsewhere,^34^ we found that there has been an exponential growth in research on the topic of peer support, from no publications prior to 2000 to a steady stream of studies since 2010. Similarly, across both sets of studies, a geographical aspect was evident, with most studies being conducted in the U.S., the fewest taking place in Europe, and a large number of studies being conducted in low-resource settings. A setting-specific approach acknowledges that low-resource and high-resource settings have different needs, which is evident in the context of studies. With respect to the participant characteristics, an approximately equal number of men and women were included, and other priority groups were people who inject drugs, men who have sex with men, people of color, and individuals with little disposable income. Although this suggests a varied priority population, the low number of studies that included non-binary genders is noteworthy. This was true despite the increased risk of acquiring HIV infection among these individuals compared to the general population.^2^

Taken together, our two reports of empirical research on peer support for PLHIV, despite our relatively narrow inclusion criteria, show that 17 different labels are being applied, with “peer” and “peer counsellors” being the most frequently used. In combination with other terms related to the specific role of “peer” support, peers may be the most flexible label, suitable for various interventions and functions, and corresponds to the key functions described by Fisher et al.^16^ and the Peers for Progress program. Similarly, the versatility in the practice of peer support found in our scoping review confirms peer support as a flexible approach to outreach that can be adapted to different settings. Still, there seem to be benefits in ensuring an understanding of both the characteristics and key functions of peer supporters. Our analysis of the key functions of peer support in the included studies demonstrates that most of the interventions combine several key functions that align with HIV as a CLLC. Although none of the included studies explicitly focused on ongoing support related to a CLLC as a key function, the many-faceted interventions indicated otherwise.

In contrast to the plentiful examinations of the effects of peer support,^34^ few studies have examined experiences with peer support for PLHIV from the providers’ perspective and still fewer from the receivers’ perspective. A similar observation was recently made in a related review.^25^ The experiences described in the included studies substantiate the idea that peer supporters contribute as role models among PLHIV. Related studies examining the receivers’ perspective show that meeting a peer supporter builds various types of support and connections to the wider community. Thus, social support from peers may be a resource when people experience stress in response to stigma.^17,19,20^ Notably, the experiences described from the perspective of the receivers of peer support are only described in four studies that reflect participants from the U.S. and Canada.

Despite the existence of only a handful of studies covering program descriptions and training of peer supporters, we found that the development of the role of peer supporters was deliberated in several settings. According to both the Australian and the UK HIV Peer Support Standards, peer support should be provided *to* PLHIV *by* PLHIV, and the peer support description and function should be tailored to the needs of specific populations.^12,18^ Our results indicate a positive awareness of the peer supporter role, quality, and function supported in this review.

The increased number of publications on peer support for PLHIV over the last decade has shown a growing interest in this topic. Despite this, we recognize the need for more studies in Europe, sub-Saharan Africa, and Russia. Few of the included studies were conducted in sub-Saharan Africa, a region with a high prevalence of HIV that has been identified by the WHO as having a vulnerable and at-risk population,^3^ and we identified no studies from Russia, which is one of only a few countries with increasing HIV incidence rates.^3^

Our results argue for a broader scope when the experiences of peer support are examined from the perspectives of providers and receivers regarding living with HIV as a CLLC.^11,16^ The Global Health Sector Strategy on HIV 2016 to 2020 recommends an integrated care package designed to meet people's needs and preferences and increase self-management related to CLLC. There is a need to clarify the support needed by PLHIV as individuals living with CLLC. Our results highlight the fact that peer support can provide practical, informational, emotional, and social support, and specifically help shoulder existing services, which is supported by other reviews.^22,23^ Despite the feeling of being empowered and gaining self-awareness, it is worth noticing the work-related stress peer supporters are addressing. The results demonstrated a scarcity of studies that include experiences from peer supporters and recipients, which is a perspective that healthcare entities should consider when improving their services. Therefore, our results are relevant for policymakers and healthcare providers to continue developing peer support programs and training of peer supporters to the specific needs of PLHIV. Further, the included studies highlight the need for quality peer support training followed by increased role clarity when integrating peer support into healthcare services.

### Strength and limitations

The systematic approach regarding searches, selection, and data extraction is the main strength of our scoping review. However, a limitation is the absence of studies in languages other than English. Nevertheless, the charting and analyses of the data made it possible to identify and maintain consistency for all categories. Another limitation was that the included studies had several labels for peer supporters previously unknown to the researchers. This could have affected the search strategy, and we might have missed some relevant studies.

## Conclusions

Research on peer support for PLHIV has increased in the last decade. This is not surprising given the increased life expectancy of PLHIV following the introduction of ART; hence, peer support has become a more integrated part of healthcare services. However, this scoping review revealed gaps in the evidence emanating from research. There is also a need for more studies related to the *experiences* of receiving peer support, *training* of peer supporters, and *program descriptions,* particularly in Europe, sub-Saharan Africa, and Russia. With about 25.4 million people accessing ART,^1^ there is an increasing need for support related to retention in care and chronic care. The increased need for setting specific peer support programs and role clarity has implications for further research. The flexibility of the peer support role related to settings and populations appears to complement healthcare services concerning the different needs of PLHIV.

## Supplemental Material

sj-DOC-1-jiapac-10.1177_23259582211066401 - Supplemental material for A Scoping Review of the Empirical Literature on Peer Support for People Living with HIV Click here for additional data file. Supplemental material, sj-DOC-1-jiapac-10.1177_23259582211066401 for A Scoping Review of the Empirical Literature on Peer Support for People Living with HIV by Anita Øgård-Repål, Rigmor C. Berg and Mariann Fossum in Journal of the International Association of Providers of AIDS Care (JIAPAC)

sj-DOC-2-jiapac-10.1177_23259582211066401 - Supplemental material for A Scoping Review of the Empirical Literature on Peer Support for People Living with HIV Click here for additional data file. Supplemental material, sj-DOC-2-jiapac-10.1177_23259582211066401 for A Scoping Review of the Empirical Literature on Peer Support for People Living with HIV by Anita Øgård-Repål, Rigmor C. Berg and Mariann Fossum in Journal of the International Association of Providers of AIDS Care (JIAPAC)
